# Integration in the light of curriculum design

**DOI:** 10.3205/zma001287

**Published:** 2019-11-15

**Authors:** Marjo Wijnen-Meijer

**Affiliations:** 1 Technical University of Munich, School of Medicine, TUM Medical Education Center, Munich, Germany

## Editorial

When discussing the curriculum, it is often the content that is the main topic of discussion.

- Which topics should be addressed?
- What skills should be learned?
- What concepts should a student learn?

Of course, the content of the training is important, but also the structure is relevant. Because new knowledge is built on existing knowledge, the order in which something is learned affects how the student ultimately remembers what he has learned. 

An important concept when it comes to the structure of a curriculum is integration.

The first form of integration is horizontal integration, whereby different disciplines, traditionally taught at the same stage of the curriculum, are thematically clustered. An example of this is a system-based curriculum. In such a curriculum, all relevant knowledge about, for example, the cardiovascular or musculoskeletal system is taught during the same period [1]. 

Although this form of integration makes it easier for students to make the connections between different subjects, the distinction between the pre-clinical (theoretical) and clinical (practical) phase remains. This often results in difficulties for students to apply the knowledge they have learned in the preclinical phase in practice [2]. 

In order to facilitate the transition from the pre-clinical phase to the clinical phase, many curricula at medical schools are structured according to the principle of vertical integration. This principle is also described in the Masterplan Medizinstudium 2020, with the aim to connect the clinical and theoretical content. 

This form of integration involves the integration of basic science and clinical practice, with both aspects being programmed throughout the curriculum [3]. Another important feature of vertical integration is that students gain clinical experience in clerkships or other forms of contact with patients from the very beginning of medical school [4], [5]. Research shows that early clinical experience leads to increased motivation and improvement of clinical skills [5], [6]. In addition, the students have a better understanding of the relevance of the theory and they learn early on what it means to behave professionally and to work in a team [5], [6].

A central part of vertical integration is the combination of the theoretical and the practical part of the programme. Both components are necessary for the development of medical expertise, and the learning effect is greater if the connection between theory and practice is clear to the students [7], [8]. By applying the theory in practice, the theory gets more meaning, which makes it easier to really understand it, which makes it more applicable in practice the next time.

In addition, when students come into contact with specific patient cases early in the curriculum, they build mental structures to which clinical information and pathologies can be linked. These so-called illness scripts make it easier for students to diagnose and to work in clinical practice [9].

Another important part of vertical integration is the increased role of workplace learning. This gives students the opportunity to perform authentic activities in a real-life context [10], [11]. By gradually phasing out supervision and giving them more responsibility, they are progressively being prepared for their future work as doctors. As early as 1978, Vygotsky described in his theory on the “Zone of Proximal Development” that competence development should take place step by step, with the help of a more experienced person. When a trainee performs a task for the first time, the supervisor has full control and responsibility. This external control decreases gradually, until the trainee no longer needs it at all and can carry out the task completely independently [12]. 

In addition, workplace learning they learn how to work in a team and are included in the medical community at an early stage [13]. In 1991, Lave and Wenger introduced the term “Community of Practice”. This indicates that it is important for learners to be part of a professional community. The cooperation between inexperienced and experienced members stimulates the exchange of knowledge. In the beginning, the new members fulfil the role of “peripheral” participants and are increasingly shifting towards core participants, whereby they can in turn guide new members [14].

Although vertical integration concerns the structure of medical training, the discussion should not focus on the distribution of hours for certain parts of the curriculum. The core of vertical integration is that students have sufficient and early contact with clinical practice, combined with a good theoretical basis. All this should help to ensure that students are well prepared for their work as doctors, both in terms of knowledge and skills, as well as functioning as a member of the community. This issue describes a number of topics and examples that fit in well with this model.

In Trauschke's article [15] it becomes clear that it is not self-evident that what the students learn in the pre-clinical phase can actually be applied in the clinical phase. There appear to be misconceptions regarding the Physiological Electrocardiogram. Kühl et al. [16] describe a very practical example of vertical integration through the improvement of a Biochemistry Course. Engel et al. [17] also describe how the connection between example cases and real patient cases can be made, during a course in general medicine. The articles by Kadmon et al. [18] and Rotthoff et al. [19] describe how workplace learning can be better structured in the practical year. Through the introduction of entrustable professional activities and structural feedback sessions, students are gradually prepared for their future work. Gebhard et al. [20] report in their article how clinical experience and early contact with the various disciplines have an influence on the final specialization choice of students. When preparing for work as a doctor, teaching is also relevant. And Fröhlich et al. [21] describe how student tutors can be prepared to conduct an ultrasound training to younger students. This allows them to better fulfil their role as a core participant in the community of practice.

We hope these articles will inspire curriculum developers in the further design of medical education.

## Competing interests

The author declares that she has no competing interests.

## References

[1] Harden RM, Sowden S, Dunn WR (1984). Educational strategies in curriculum development: the SPICES model. Med Educ.

[2] Prince KJ, Boshuizen HP, van der Vleuten CP, Scherpbier AJ (2005). Students's opinions about their preparation for clinical practice. Med Educ.

[3] Prideaux D, Dent JA, Harden RM (2009). Integrated learning. A practical guide for medical teachers.

[4] Wijnen-Meijer M, ten Cate OT, van der Schaaf M, Borleffs JCC (2010). Vertical integration in medical school: effect on the transition to postgraduate training. Med Educ.

[5] Kamalski DM, Braak EW, Cate OT, Borleffs JC (2007). Early clerkships. Med Teach.

[6] Dornan T, Bundy C (2004). Learning in practice: What can experience add to early medical education? Consenses survey. BMJ.

[7] Regehr G, Norman GR (1996). Issues in cognitive psychology: implications for professional education. Acad Med.

[8] Dreyfus HL, Dreyfus SE (1984). Mind over machine: The power of human intuition and expertise in the era of the computer.

[9] Torre DM, Hernandez DA, Castiglioni A, Durning SJ, Daley BJ, Hemmer PA, LaRochelle J (2019). The Clinical Reasoning Mapping Exercise (CResME): a new tool for exploring clinical reasoning. Perspect Med Educ.

[10] Liljedahl M (2018). On learning in the clinical environment. Perspect Med Educ.

[11] Ramani S, Leinster S (2008). AMEE Guide no. 34: Teaching in the clinical environment. Med Teach.

[12] Vygotsky LS (1978). Minds in society. The development of higher psychological processes.

[13] Dornan T, Boshuizen HP, King N, Scherpbier AJ (2007). Experience-based learning: A model linking the processes and outcomes of medical students'workplace learning. Med Educ.

[14] Lave J, Wenger E (1991). Situated learning: legitmate peripheral participation.

[15] Trauschke M (2019). A qualitative study on the development and rectification of advanced medical students' misconceptions about the physiological electrocardiogram (ECG). GMS J Med Educ.

[16] Schneider A, Kühl M, Kühl S (2019). Longitudinal curriculum development: gradual optimization of a biochemistry seminar. GMS J Med Educ.

[17] Engel B, Esser M, Bleckwenn M (2019). Pilotin a blended-learing-concept for integrating evidence-based medicine into the general practice clerkship. GMS J Med Educ.

[18] Berberat PO, Rotthoff T, Baerwald C, Ehrhardt M, Huenges B, Johannink J, Narciss E, Obertacke U, Peters H, Kadmon M (2019). Entrustable professional activities in final year unterdraduate medical training. GMS J Med Educ.

[19] Schick K, Eissner A, Wijnen-Meijer M, Johannink J, Huenges B, Ehrhardt M, Kadmon M, Berberat PO, Rotthoff T (2019). Implementing a logbook on Entrustable professional activities in the final year of undergraduate medical education in Germany - a multicentric pilot study. GMS J Med Educ.

[20] Gebhard A, Müller-Hilke B (2019). Criteria of medical students fort he selection of their future clinical specialisation: a cross-sectional survey at the Medical Faculty of Rostock. GMS J Med Educ.

[21] Celebi N, Griewatz J, Ilg M, Zipfel S, Riessen R, Malek NP, Pauluschke-Fröhlich J, Debove I, Muller R, Fröhlich E (2019). Three different ways of training ultrasound student-tutors yield significant gains in tutee's scanning-skills. GMS J Med Educ.

